# Five years calibrated observations from the University of Bonn X-band weather radar (BoXPol)

**DOI:** 10.1038/s41597-022-01656-0

**Published:** 2022-09-08

**Authors:** Velibor Pejcic, Joshua Soderholm, Kai Mühlbauer, Valentin Louf, Silke Trömel

**Affiliations:** 1 Institute of Geosciences, Department of Meteorology, University of Bonn, Bonn, Germany; 2 Bureau of Meteorology, Melbourne, Australia; 3 Laboratory for Clouds and Precipitation Exploration, Geoverbund ABC/J, Bonn, Germany

**Keywords:** Physics, Atmospheric science

## Abstract

Polarimetric weather radars offer a wealth of new information compared to conventional technology, not only to enhance quantitative precipitation estimation, warnings, and short-term forecasts, but also to improve our understanding of precipitation generating processes and their representation in numerical weather prediction models. To support such research opportunities, this paper describes an open-access dataset between 2014–2019 collected by the polarimetric Doppler X-band weather radar in Bonn (BoXPol), western Germany. To complement this dataset, the technical radar characteristics, scanning strategy and the best-practice for radar data processing are detailed. In addition, an investigation of radar calibration is presented. Reflectivity measurements from the Dual-frequency Precipitation Radar operating on the core satellite of the Global Precipitation Mission are compared to those of BoXPol to provide absolute calibration offsets with the dataset. The Relative Calibration Adjustment technique is applied to identify stable calibration periods. The absolute calibration of differential reflectivity is determined using the vertical scan and provided with the BoxPol dataset.

## Abstract (summary)

**Table:** 

Measurement(s)	Radar backscattering of precipitation
Technology Type(s)	Polarimetric Doppler X-band weather radar

## Background & Summary

This paper describes a 66 months (5.5 years) dataset of polarimetric measurements and related calibration data from the Dual-Pol X-Band radar operated by the University of Bonn, Germany (BoXPol). BoXPol is connected to the Jülich Observatory for Cloud Evolution (JOYCE^1,2^) forming the infrastructure of the Clouds and Precipitation Exploration Laboratory, i.e. the competence centre of the geoscientific network of the Aachen-Bonn-Cologne/Jülich research area. Overlapping with the national polarimetric C-band radar network of the German Weather Service (DWD), BoXPol serves amongst others as a database in research programs like the special priority program on *Fusion of Radar Polarimetry and Numerical Atmospheric Modelling Towards an Improved Understanding of Cloud and Precipitation Processes*^3,4^, the research unit on *Near-Realtime Quantitative Precipitation Estimation and Prediction*^5^ and the *Hans Ertel Centre for Weather Research* (HErZ^6^). *The Collaborative Research Centre*/*Transregio 32*^7,8^ aimed at the development of a holistic view of the terrestrial system and identified the Rur catchment, covered by BoXPol, as its central observation site because of its strong diversity with respect to weather, soil types, and land use. Thus, BoXPol plays a key role in meteorological research and teaching at the institutes involved. BoXPol observations provide deep insights into atmospheric dynamics and microphysical processes of precipitation across warm and cold seasons in the regional temperate climate of western Germany^9,10^. Measurements from this radar have been exploited for in-depth microphysical evaluation of the Icosahedral Nonhydrostatic (ICON) atmospheric model in LES configuration^11–13^, and to study the polarimetric signatures of size sorting^14^, freezing^15^, riming and aggregation^16,17^. Furthermore, BoXPol measurements provided insights into the quantification and information content of backscatter differential phase^18^, polarimetric characterization of microphysical processes in the melting layer^19^ and the quantification of evaporation and cooling rates^20^. Combined with other sensors, the BoXPol dataset has been exploited for an in-depth analysis of mammatus clouds^21^. BoXPol measurements have been employed to introduce the new polarimetric rainfall retrieval technique based on specific attenuation^22^, to analyze hail events with combined dual-Doppler and polarimetric information^23^, to investigate snow retrievals and nowcasting applications based on signatures in the dendritic growth layer^10^ and to demonstrate the benefit of radar-based rainfall retrievals for flood prediction^24,25^. However, careful quality control, calibration, and processing is a mandatory prerequisite for the scientific exploitation of polarimetric radar data. Therefore, calibration offsets and best-practice processing scripts that utilise libraries from wradlib^26^ and Py-ART^27^ are provided with the BoXPol dataset.

Section *Methods* of this paper includes the technical description and scan strategy of the polarimetric X-band radar in Bonn (BoXPol), while Section *Technical Validation* outlines the calibration of horizontal reflectivity and differential reflectivity and the recommended data processing and correction algorithms for ground-based radar observations. An overview of the archive and the data formats is presented in Section *Data Records*.

## Methods

**Fig. 1:**
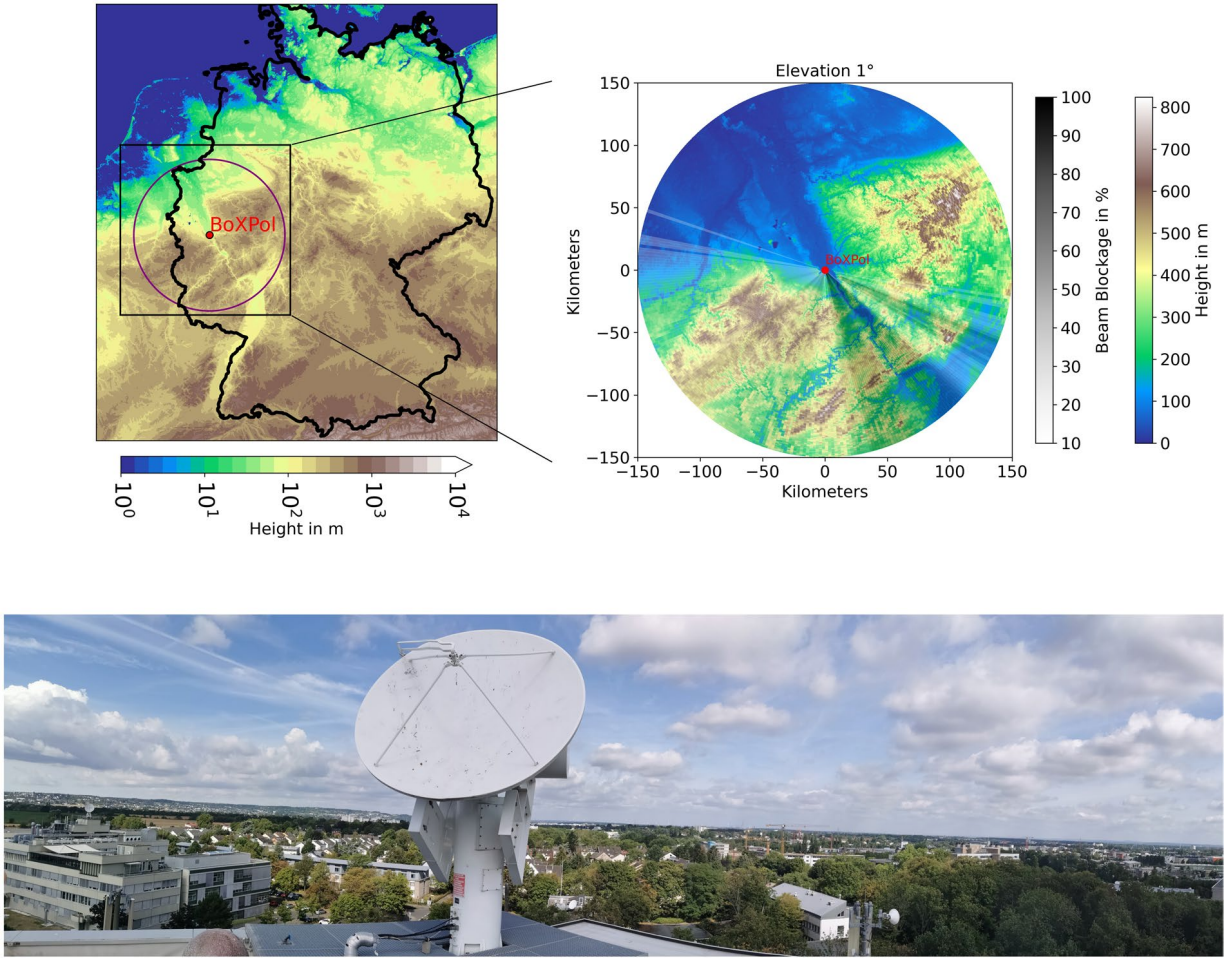
Top left: Location and coverage of the BoXPol weather radar in Bonn, Germany (blue circle). Top right: Zoom in the Bonn region with dark shaded areas experiencing significant beam blockage in the 1° Plan Position Indicator scan. The location of the radar is marked with a red dot. Bottom: BoXPol weather radar at the top of a 30 m high building next to the Meteorological Department in Bonn.

The BoXPol weather radar is located in Bonn (50.7305° North and 7.0717° East), Germany, at 99.5 m above mean sea level (Fig. 1) on the rooftop of a 30 m building next to the department of meteorology of the University of Bonn. The hardware consists of a radome-less EEC DWSR-2001-X-SDP weather radar operating in Simultaneous Transmit and Receive of H and V channels (STAR) mode using an Enigma signal processor (Enigma 3 upgraded to Enigma 4 in April 2017). The random-phase magnetron system operates at a frequency of 9.3 GHz and employs a scanning strategy consisting of ten different plan position indicator (PPI) scans with elevation angles between 1° and 28°, a birdbath scan (90°) and a range-height indicator (RHI) scan within a 5 minutes scan schedule (approximately 30 s per scan). The technical characteristics are displayed in Tables 1, 2 summarizes elevation angle, maximal range, range resolution and the pulse repetition frequency (PRF) for all PPI scans with Enigma 3 and 4, respectively, i.e. before and after April 2017. The azimuthal resolution is 1° while the range resolution depends on the scan configuration (Table 2) and varies between 25 and 150 meters. The lowest PPI measured at 1° covers 150 kilometers range. A beam-blockage map and its derivation based on specific attenuation is provided in^28^.

**Table 1:** Technical description of the X-band radar BoXPol.

	Specification
Location	Bonn (Germany)
Latitude	50.7305° N
Longitude	7.0717° E
Altitude	99 m
3-dB beamwidth	1°
Signal processor	GAMIC Enigma 3/4
	*changed 2017-04-03*
Temporal resolution	5 min
Number of PPI scans	10
Special scans	RHI and birdbath
Elevation angels (PPI)	1° to 28°
Azimuth angels (PPI)	1° to 360°
Maximum range	150 km
Radial resolution	25 m to 200 m
Transmit type	Dual-Pol STAR

**Table 2:** Scanning strategy configuration for all elevations of the BoXPol scan for Enigma 3 and Enigma 4.

Elevation [°]	2014-01-01 - 2017-04-03			2017-04-04 - 2019-06-30		
	Range [km]	Enigma 3	PRF [Hz]	Range [km]	Enigma 4	PRF [Hz]
		Resolution [m]			Resolution [m]	
1.0	150	150	400	150	200	700
1.5	100	100	950	—	—	—
2.0	—	—	—	150	200	800
2.4	100	100	1000	—	—	—
3.1	—	—	—	150	200	900
4.5	100	150	950	150	200	950
6.0	—	—	—	140	150	1050
7.0	50	25	1000	—	—	—
8.2	110	100	1150	100	125	1150
11.0	100	100	1150	80	125	1150
14.0	80	100	1150	62	125	1150
18.0	55	100	1150	50	125	1150
28.0	35	100	1150	36	125	1150

## Data Records

**Fig. 2:**
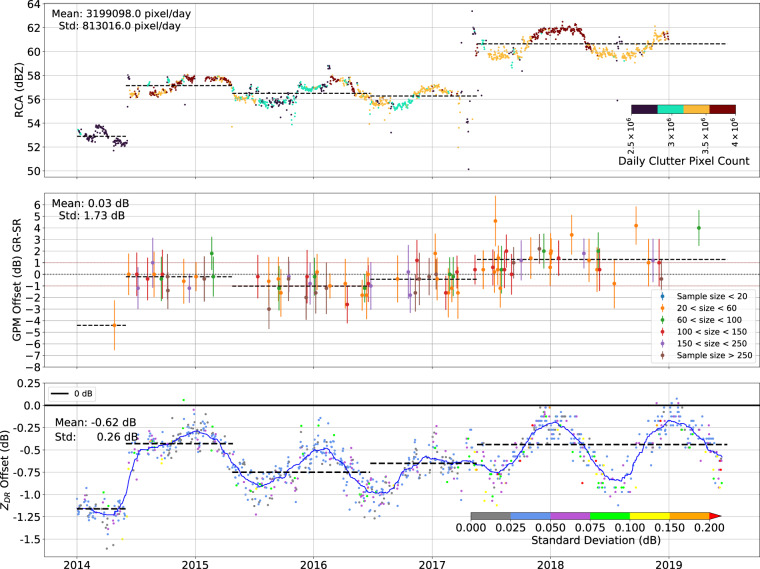
Top: Time series of the daily average 95th percentiles of clutter reflectivity. The dashed black lines represent the base line for the selected periods. The colors indicate the daily clutter pixel counts. Middle: Time series of the matched space borne (SR) and ground based radar (GR) reflectivity differences between 2014 and 2019. The colors indicate the number of matched samples and the error bar shows the standard deviation of the reflectivity differences. The dashed black lines represent the mean of all matched points within the chosen time periods. Bottom: Time series of *Z*_*DR*_ offsets derived with the birdbath method. The blue line is the moving mean with a 3 months window size and the dashed black lines represent the mean of all points within the chosen time periods. The colors represent the standard deviation of the daily *Z*_*DR*_ offset.

The archive dataset consists of daily netCDF files (Conventions CF-1.7 (https://github.com/cf-convention/cf-conventions) and following Cf/Radial-2.1 (no standard yet)) for each of the ten PPI scans (birdbath scan and RHI will be included in later versions) and includes the following polarimetric variables: reflectivity at horizontal polarization (*Z*_*H*_), reflectivity at vertical polarization (*Z*_*V*_), differential reflectivity (*Z*_*DR*_), cross-correlation coefficient (*ρ*_*hv*_), total differential phase (Φ_*DP*_), uncorrected horizontal/vertical reflectivity factor (*T*_*H*_, *T*_*V*_), horizontal/vertical radial velocities (*V*_*H*_, *V*_*V*_) and horizontal/vertical spectral width of radial velocity (*W*_*H*_, *W*_*V*_). Note that *Z*_*H*_ and *Z*_*V*_ are corrected for clutter, speckle, interference and second/third trip echoes by the radar processor. In relation to these corrections, a clutter map (CMAP) is also available since April 2017. Calibration offsets (see Fig. 2 and Table 3), however, need to be applied by the user. The data is archived by the DKRZ (German Climate Computing Centre^29^)

**Table 3:** Calibration offsets for BoXPol’s horizontal reflectivity (*Z*_*H*_) and differential reflectivity (*Z*_*DR*_) in the selected periods.

Start	End	*Z*_*H*_ offset [dB]	*Z*_*DR*_ offset [dB]
2014-01-01	2014-05-31	−4.40 ± 2.15	−1.16 ± 0.05
2014-06-01	2015-04-24	−0.21 ± 1.78	−0.44 ± 0.14
2015-04-25	2016-06-23	−1.02 ± 1.76	−0.75 ± 0.14
2016-06-24	2017-05-18	−0.43 ± 1.73	−0.67 ± 0.15
2017-05-19	2019-06-30	1.28 ± 1.66	−0.47 ± 0.21

## Technical Validation

### Processing of ground based radar data

Accurate absolute calibration of radar data requires a thorough preprocessing. Even though raw data is provided, the algorithms we applied before the calibration are outlined in the following as an optional guideline. First, the BoXPol polarimetric moments are filtered for erroneous observations by excluding reflectivities *Z*_*H*_ lower than −20 dBZ and higher than 80 dBZ, differential reflectivities *Z*_*DR*_ lower than −6 dB and higher than 7 dB, differential phase texture SD(Φ_DP_) higher than 20° ^30^ and cross-correlation coefficient *ρ*_*hv*_ lower than 0.6 to remove non-meteorological signals. The SD(Φ_DP_) is the spatial variability of Φ_*DP*_, expressed as the root mean square difference in a region of three pixels in range and azimuthal direction. This variable is e.g. used in^30^ to distinguish between precipitating and non-precipitating echoes. We follow^31^ to process raw Φ_*DP*_ with linear programming to provide improved estimates of Φ_*DP*_, in the following referred to as processed Φ_*DP*_, and to derive specific differential phase *K*_*DP*_ (Py-ART^27^,). *K*_*DP*_ values lower than −4° km^−1^ and higher than 15° km^−1^ are excluded from the dataset. In the ensuing step processed Φ_*DP*_ is used for attenuation correction using the ZPHI method^32^. The correction is only applied to the liquid region below the freezing level determined with the ERA5 geopotential height and dry bulb temperature profiles on pressure level dataset^33^ following^34^. Linear interpolation was applied to get the geopotential height exactly at the 0 °C level. Based on the 3 second resolution Digital Elevation Model (DEM) from NASA’s Shuttle Radar Topography Mission^35^, the method from^36^, implemented in the wradlib library^26^, is applied to determine partial beam-blockage (PBB). Areas showing PBB >10% are excluded to improve the accuracy of calibration retrievals. For example Fig. 1 illustrates affected areas for the PPI at the lowest (1°) elevation angle^37^.

### Calibration of horizontal reflectivity

We applied the relative calibration adjustment (RCA) technique to determine stable calibration periods and also volume matching with a satellite-based precipitation radar of the Global Precipitation Mission Core-satellite (GPM^38,39^) for the absolute calibration. In contrast to conventional calibration methods^40–43^, these two calibration techniques do not require any changes with the operational scan strategy or extra hardware installations. Furthermore^39^, demonstrated that the use of the self-consistency technique for calibration purposes as described in^44^ requires additional local disdrometer measurements to determine the relationship between *K*_*DP*_/*Z*_*H*_ and *Z*_*DR*_. Without this assumption the difference between characteristic drop size distributions in the mid-latitudes used in^45^ and the tropical case in^39^ led to 2 dB difference in calibration. The Dual-frequency Precipitation Radar (DPR) observations are well-calibrated using internal and external calibration^38^ with an accuracy within ±1 dB^46^ and the satellite-based measurements are freely available (https://storm.pps.eosdis.nasa.gov/storm/). The RCA technique can be applied continuously even in absence of precipitation.

#### Relative calibration

The RCA method exploits statistics generated from local stable clutter^39,47^ to detect changes in calibration offsets. Radar pixels within 20 km range of the lowest scan are identified as stable clutter if the uncorrected reflectivity is 50 dBZ or higher in at least 50% of the daily measurements. The 95th percentile of all reflectivity samples within the persistent clutter bins is then used to estimate the relative calibration for that day. Application of RCA to the BoXPol data set reveals four significant changes in calibration across the period with GPM overpasses, namely on 2014-06-01, 2015-04-25, 2016-06-24 and 2017-05-19 (Fig. 2). Indeed, radar hardware changes, operational changes or radar services occurred on these dates, which confirms the reliability of the method. For each stable period identified between two subsequent changes in the RCA time series (Fig. 2, top), the GPM radar measurements (more details on the GPM measurements are provided in section ‘Absolute Calibration’) are used to determine the respective mean absolute calibration values (Fig. 2, center). The RCA time series is not sufficiently stable to provide relative calibration based on the mean GPM offset for each period, as recommended by^39^. Rather, the RCA time series shows strong seasonal variability, with increased values during warmer and decreased values during cooler months. Therefore we use the RCA time series only to select stable periods to use for calibration with GPM and do not apply the RCA analysis for calibration. The overall mean and standard deviation of the daily stable clutter pixels used for the relative calibration is also indicated in Fig. 2 (top). We hypothesize these seasonal variations are the result of the annual temperature cycle, however, similar findings have not been documented before and further investigations are suggested to corroborate this connection.

#### Absolute calibration

Due to lower attenuation compared to *K*_*α*_-band, this study exploits the *K*_*u*_-band (13.6 GHz) measurements of the DPR on board of the Global Precipitation Mission (GPM) for calibration of the ground-based radar BoXPol. The *K*_*u*_ system has a footprint of 5 km, 125 m vertical resolution and 245 km swath width^48,49^. GPM overpasses for Germany occur approximately twice per day and we selected all overflights in the period from 8 August 2014 (first rain event in BoXPol area after GPM launch) to 8 April 2019 with more than 1% of the BoXPol region covered with precipitation. This region is defined between 51.4° and 49.4° north and 9.0° and 5.8° east. The GPM data (version 5, file specification 2AKu) are freely available. Specific GPM parameters required for the calibration technique are the quality index (dataQuality), zenith angle (localZenithAngle), precipitation flag (flagPrecip), bright band height (heightBB), bright band width (widthBB), bight band quality (qualityBB), precipitation type (typePrecip), precipitation type quality (qualityTypePrecip) and attenuation corrected reflectivity (zFactorCorrected). For more detailed information about specific GPM parameters we refer to^38^.

**Fig. 3:**
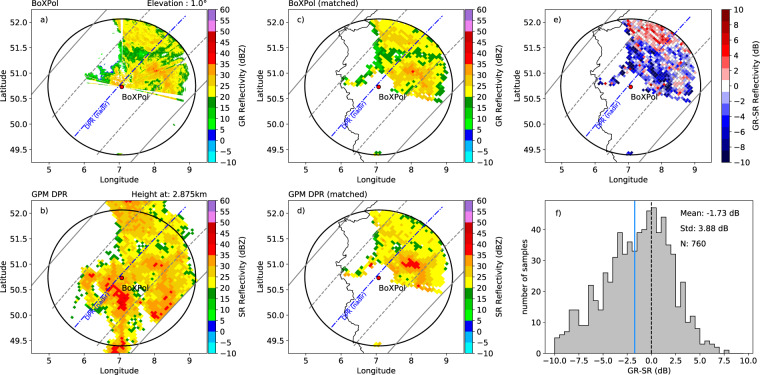
Comparison between BoXPol radar and DPR on 2014-10-07, where the nadir beam is indicated with the blue dashed line, the inner swath with the dashed gray lines and the outer swath with the solid gray lines. The reflectivities of the ground-based radar GR (panel a) and of the satellite-based radar SR (panel b), the matched GR (panel c) and SR reflectivity samples (panel d) and the GR-SR reflectivity differences (panel e) of the PPI scan measured at 1° elevation for all individual matched samples are shown. The GR-SR reflectivity difference distribution (panel f) is shown together with the mean, indicated as a blue solid line, standard deviation and number of matched samples. The black dashed line indicates a difference of 0 dB.

In this technique, the *K*_*u*_-band radar bins of the space-borne radar (SR, Fig. 3b) are geometrically matched with the radar beams of the ground-radar (GR, Fig. 3a) to enable the comparison of identical volumes. Therefore all BoXPol bins located in a DPR footprint and all DPR bins from the same footprint located vertically within the BoXPol radar beam are identified and averaged (matched). The averaged reflectivities of the GR bins corresponding to the DPR footprints are shown in Fig. 3c and the averaged reflectivities of the SR bins corresponding to a vertically GR beam width are shown in Fig. 3d. The generated matched volumes are used for the calibration (see Fig. 3c/d, more details on the matching are shown in Fig. 2 of^38^). The *Z*_*H*_ offset is calculated by subtracting the SR reflectivity from the GR reflectivity (Fig. 3e) followed by averaging over all matched samples identified in one overpass (Fig. 3f). In order to take differences between the frequencies into account, the *K*_*u*_-band reflectivity (*Z*_*H*_(*K*_*u*_)) is first converted to X-band (*Z*_*H*_(*X*)) following mainly the S-*K*_*u*_ band conversion introduced by^50^. We performed T-matrix scattering simulations^51^ for rain, dry snow and dry hail to simulate the reflectivities at *K*_*u*_ and X-band. Drop size distributions, particle orientation, the complex dielectric constant and the aspect ratio are simulated as in^50^. We calculated the aspect ratio for snow following^52^ and for hail following^53^ and the dielectric constant is calculated according to^54^. Thus, to convert the SR measured at *K*_*u*_-band to X-band the following equation is applied:

1 $${Z}_{H}\left({\rm{X}}\right)={Z}_{H}\left({{\rm{K}}}_{{\rm{u}}}\right)+\mathop{\sum }\limits_{i=0}^{5}{c}_{i}{\left[{Z}_{H}\left({{\rm{K}}}_{{\rm{u}}}\right)\right]}^{i}.$$

The last term is the dual-frequency ratio with the specific coefficients for the frequency conversion *c*_*i*_ in rain, dry snow and dry hail are provided in Table 4. The overall accuracy of the frequency conversion is 0.23 dB for rain, 0.42 dB for dry snow and 0.20 dB for dry hail. Note that the transformation for hail is only used if the DPR has detected convective precipitation above the bright band.

**Table 4:** Coefficients for relationship to convert reflectivities at *K*_*u*_-band to X-band. The coefficients are given for rain, dry snow and dry hail.

	*c*_0_	*c*_1_	*c*_2_	*c*_3_	*c*_4_	*c*_5_
Rain	1.91 × 10^−1^	−7.83 × 10^−2^	1.12 × 10^−2^	−6.17 × 10^−4^	1.25 × 10^−5^	−8.43 × 10^−8^
Dry snow	−1.2 × 10^−1^	6.80 × 10^−2^	−4.55 × 10^−3^	1.18 × 10^−4^	−6.60 × 10^−7^	0
Dry hail	5.57 × 10^−2^	−1.80 × 10^−2^	1.91 × 10^−3^	−6.64 × 10^−5^	8.18 × 10^−7^	0

**Fig. 4:**
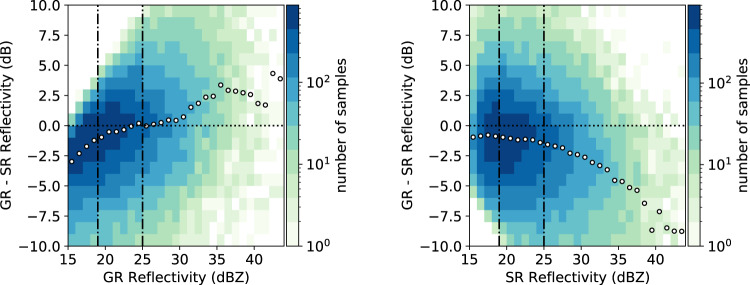
GR - SR difference as a function of GR reflectivity (left) and SR reflectivity (right) displayed in a two-dimensional histogram for all matched volumes between 2014 and 2019. White dots indicate the median GR - SR differences per 1 dBZ bin. Colors indicate the number of samples and the vertical dashed lines the selected reflectivity thresholds for the calibration offset calculation.

GPM overpasses containing at least 10 valid precipitation samples (indicated by the flagPrecip fields in GPM files) within 20 to 150 km range from the ground radar site have been selected for the comparison with the BoXPol dataset. Searching for the closest radar volume in time for each GPM overpass a maximum time difference of 2.5 min between the BoXPol volume start time and the GPM overpass time was allowed. Following^38^, we verified the sensitivity of the GR-SR difference to the GR (Fig. 4, left) and SR (Fig. 4, right) reflectivities for all matched volumes and all overpasses to identify the reflectivity thresholds for the calibration. Only reflectivities between 19 dBZ and 25 dBZ have been taken into account. The upper threshold mitigated the impact of uncertainties in the attenuation correction of DPR measurements and the lower threshold is due to SR sensitivity. Matched samples with standard deviations greater than 4 dBZ or observations contaminated with bright band are removed. The minimum number of pairs in one matched volume is set to 20. With these constraints, 85 valid DPR overpasses for liquid and solid precipitation in the BoXPol region have been identified for the dataset. Absolute *Z*_*H*_ offsets for periods identified as stable with the RCA are provided in Table 3 with standard deviations ranging between 1.66 dB and 2.15 dB. Similar deviations can be found in^39^ and^37^. The overall mean calibration offset and standard deviation are 0.03 and 1.73 dB (see also Fig. 2, center). Note that the calculated reflectivity calibration offset in Fig. 2 (center) have to be subtracted from the ground-based radar reflectivity.

### Calibration of differential reflectivity

Calibration time series for differential reflectivity *Z*_*DR*_ are determined using the measurements in light precipitation with the birdbath scan at 90 deg elevation^55,56^. Since the mean canting angle of small raindrops is close to 0°, they appear spherical seen from below, which implies that both the *Z*_*V*_ and *Z*_*H*_ are expected to be equivalent and deviations from *Z*_*DR*_ = 0 dB can be exploited for calibration. According to^57^ this can be applied to all hydrometeor classes. For our study we consider all regions except the melting layer. Azimuthal averaging has been performed to reduce noise and measurement within the first 600 meters have been excluded due to possible clutter contamination. To avoid any biases introduced by strong precipitation events and melting particles, only samples with *Z*_*H*_ < 30 dBZ and *ρ*_*HV*_ > 0.99 have been included in the analysis. All data 250 meters above and below the freezing level are also removed. Here, the freezing level height derived from the ERA5 reanalysis is used again. To exclude turbulence, only fall velocities below 1 ms^−1^ are allowed. The median of remaining data points between the 10 and 90 percentile provides the *Z*_*DR*_ offset (^57–59^, Fig. 2, bottom). Note that the calculated *Z*_*DR*_ calibration offset illustrated in Fig. 2 and listed in Table 3 have to be subtracted from the measurements. The overall standard deviation of the *Z*_*DR*_ offset is 0.26 dB. The standard deviations in the specific periods are within the required uncertainty range between 0.1 dB and 0.2 dB^57^. The daily standard deviations also satisfy this condition with the exception of a few specific days (red colored points in Fig. 2 bottom).

## Data Availability

The described *Z*_*H*_ calibration and correction procedures are available in the python packages wradlib^26^, Py-ART^27^, cluttercal (https://github.com/vlouf/cluttercalhttps://github.com/vlouf/cluttercal) and gpmmatch (https://github.com/vlouf/gpmmatch) and demonstration scripts for data visualization, processing and absolute calibration are provided as part of the data repository and the published relative calibration codes are also available on github (cluttercal).
